# Identification of a 17 kDa protein that is a potentially novel antigen of lettuce‐associated respiratory allergy in farmers

**DOI:** 10.1002/iid3.1093

**Published:** 2023-11-20

**Authors:** Junya Yoshioka, Tatsuya Nagano, Reina Sekiya, Erika Yano, Naoya Hatano, Naoko Katsurada, Masatsugu Yamamoto, Motoko Tachihara, Yuichi Uno, Tatsuya Moriyama, Yoshihiro Nishimura, Kazuyuki Kobayashi

**Affiliations:** ^1^ Division of Respiratory Medicine, Department of Internal Medicine Kobe University Graduate School of Medicine Kobe Hyogo Japan; ^2^ Department of Applied Biological Chemistry Graduate School of Agriculture, Kindai University Nara City Nara Japan; ^3^ Department of Internal Medicine Integrated Center for Mass Spectrometry, Kobe University Graduate School of Medicine Kobe Hyogo Japan; ^4^ Department of Plant Resource Science Graduate School of Agricultural Science, Kobe University, Rokko Kobe Hyogo Japan

**Keywords:** allergen, IgE, immunologic tests, lettuce, occupational allergies

## Abstract

**Background:**

We have identified and reported a novel antigen, nonprotein‐specific secreted EP1‐like glycoprotein (51 kDa), for lettuce‐related respiratory allergy.

**Objective:**

We aimed to identify a novel antigen for lettuce‐related respiratory allergy that is different from epidermis‐specific secreted EP1‐like glycoprotein.

**Methods:**

Immunoblotting was performed using an immunoglobulin E‐specific antibody. The antigen‐antibody reaction was confirmed by means of enzyme‐linked immunosorbent assaying. LC‒MS/MS analysis was carried out to detect a novel protein found in sera from 3 of 13 patients with lettuce‐related respiratory allergy. Finally, we purified a novel protein from *Escherichia coli*.

**Results:**

Immunoblotting assays showed common bands of 17 kDa in the sera of 3 of 13 patients. An enzyme‐linked immunosorbent assay confirmed that the patient sera reacted with lettuce latex juice. A 17 kDa protein band that showed antigenic reactivity in 3 of 13 patient sera was identified as a kirola‐like protein by LC‒MS/MS. In addition, although we purified this protein, we failed to show the inhibitory effect.

**Conclusion:**

A 17 kDa protein that is a potentially novel antigen of lettuce‐associated respiratory allergy was identified. In further studies, we will focus on purifying this novel protein to diagnose lettuce allergy.

## Key message

- Immunoblotting analysis showed common bands at 17 kDa for lettuce latex and patient serum.
- The 17 kDa protein bands were identified as kirola‐like protein by LC‒MS/MS.
- We purified the kirola‐like protein.

## Graphical abstract

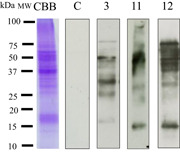

## 1 INTRODUCTION

Past reports have stated that individuals can develop occupational dermatitis after being exposed to lettuce and that systemic adverse reactions can occur after ingesting lettuce.^1^, ^2^, ^3^, ^4^, ^5^, ^6^, ^7^ In cases of oral allergy syndrome and anaphylaxis, a number of allergenic proteins have been identified, such as Lac s 1 lipid transfer protein (9 kDa),^6^, ^7^, ^8^ 16 kDa,^4^ the thaumatin‐like protein family (26 kDa), and aspartyl protease (35 and 45 kDa),^9^ these proteins being identified mainly from the leaves of lettuces.

A previous study that we carried out compared the extract from lettuce leaf juice with that from lettuce latex juice by means of immunoblotting, the results revealing noticeable differences among the immunoglobulin E (IgE)‐binding bands bound to the pooled sera. Furthermore, enzyme‐linked immunosorbent assay (ELISA) results supported the trend in which the sera of patients were more reactive with lettuce latex than with lettuce leaf extract, the negative control sera being unreactive in both cases. By using the sera of patients suffering from lettuce‐related respiratory allergies, we previously identified that the epidermis‐specific EP1‐like glycoprotein (51 kDa) secreted in lettuce latex is a lettuce‐related allergen. As previously reported, immunoblotting revealed a common band at 51 kDa in more than 50% of patients with lettuce‐associated respiratory allergy,^1^ as well as an addition common band at 17 kDa in three patients with such an allergy. We attempted identication of a novel antigen for lettuce‐related respiratory allergy by focusing on the 17 kDa band.

## 2 MATERIALS AND METHODS

### 2.1 Patients and clinical examination

Thirteen lettuce farmers having a clinical history of harvesting‐ and packaging‐related respiratory symptoms induced by lettuces took part in the study. The patients all gave informed consent in writing before participating, and the institutional review board approved the protocol (permission number #280007). The study's general parameters are available at UMIN (https://www.umin.ac.jp/) under reference number UMIN000027002.

### 2.2 Preparing the lettuce extract

**Figure 1:**
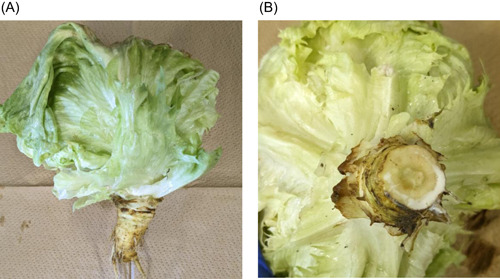
(A) Lettuce (*Lactuca sativa. var. capitata*); (B) lettuce latex.

The stem of a freshly harvested lettuce (*Lactuca sativa. var. capitata*, Figure 1A) was cut with a scalpel, and the white juice (latex) that accumulated on the section was collected (Figure 1B) and dissolved in distilled water to keep it liquified.

### 2.3 Sodium dodecyl sulfate‒polyacrylamide gel electrophoresis (SDS‐PAGE)

The lettuce protein (approximately 6–15 µg of protein per lane) was separated by means of SDS‐PAGE.^10^ To detect all of the protein patterns, Coomassie Brilliant Blue R‐350 (GE Healthcare) was used to stain the proteins in the gel.

### 2.4 Immunoblot analysis

Immunoblotting was carried out by employing a semidry blotting method to transfer the SDS‐PAGE gel to an Immobilon‐P™ polyvinylidene difluoride membrane (Millipore),^11^ a method previously described in full elsewhere.^12^ As negative controls, the sera of two nonallergic individuals were used. After incubation in PBS‐T, which contained 5% skim milk for blocking, the membranes were incubated overnight with human sera at a dilution of 1:20 at 4°C. The membranes were then washed three times with PBS‐T for 10 min and incubated with an horseradish peroxidase (HRP)‐conjugated goat anti‐human IgE antibody at a dilution of 1:3000 for 1 h. After this, the membranes were washed four times with PBS‐T for 10 min. To detect the bound secondary antibodies, an enhanced chemiluminescent Western blotting substrate (GE Healthcare) was employed, the resulting chemiluminescence being detected with X‐ray films (GE Healthcare).

### 2.5 Enzyme‐linked immunosorbent assay

The binding of patient and control serum IgE to lettuce latex was detected by means of an ELISA. To each well of the ELISA plates (Asahi Glass), 50 μL of lettuce latex in PBS at a concentration of 50 μg/mL was added, coating the plates overnight at 4°C. The plates were blocked for 1 h at room temperature with 1% BSA in 10 mmol/L PBS containing 0.1% Tween‐20 (PBS‐T) and were then washed three times with PBS‐T. Subsequently, patient or control sera at a dilution of 1:50 in Can Get Signal® Immunoreaction Enhancer Solution 1 (Toyobo Co., Ltd.) were added to the wells and incubated for 1 h at 37°C. The wells were then washed five times with PBS‐T. After this, HRP‐conjugated affinity‐purified goat anti‐human IgE antibody (Kirkegaard & Perry Laboratories) at a dilution of 1:3000 in Can Get Signal® Immunoreaction Enhancer Solution 2 was added to the wells and incubated for 1 h at 37°C. After washing five times with PBS‐T, reactions with 50 μL of a TMB peroxidase substrate (Kirkegaard & Perry Laboratories) were carried out to detect the bound secondary antibodies. The reactions were halted and the signal amplified by adding 50 μL of 1 mol/L phosphoric acid. The absorbance of each well at 450 nm was measured by means of a Wallac ARVO SX 1420 multilabel counter (PerkinElmer).

### 2.6 Mass spectrometry

Using SDS‐PAGE, the lettuce latex extracts were separated, the resulting gels were stained with Coomassie Brilliant Blue (CBB), and those bands in the same position as the common ones identified in the immunoblot in the sera of 3 of the 13 patients were extracted from the gels. LC‒MS/MS analysis was carried out by means of an LCMS‐IT‐TOF (Shimadzu) in conjunction with a nanoreverse‐phase liquid chromatography system (Shimadzu). Analysis of the MS/MS data was accomplished by means of Mascot version 2.3.01 (Matrix Science) using, as search parameters, enzyme: trypsin; variable modifications: carbamidomethyl (Cys), oxidation (Met), propionamide (Cys); peptide mass tolerance: ±0.05 Da; fragment mass tolerance: ±0.05 Da; maximum missed cleavages: 1.

### 2.7 Cloning, expression, and purification of fusion proteins

Using RT‐PCR with total RNA templates extracted from lettuce seedlings, cDNA of kirola‐like protein (XM_023895167.1) was amplified, the PCR product being directly inserted into a pColdII expression vector (Takara Bio). Appropriate PCR primers were used to express the open reading frame of the target gene and 6×His‐Tag sequence at the C‐terminal end (forwards: 5′‐AAGAGGTAATACACCATGACTTTAAGTGGTACACTA‐3′; reverse: 5′‐ATGATGATGATGATGGTTTGCTTTTGGGAGGTGA‐3′). Using an In‐fusion HD cloning kit (Becton Dickinson) in the way specified by the manufacturer, the RT‐PCR product was cloned into a pColdII vector whose full‐length cDNA nucleotide sequences were confirmed by means of a 3100 or 3130xi automated DNA sequencing system (Applied Biosystems Division, Perkin‐Elmer). Having transformed the pColdII‐kirola‐like protein construct into BL21 (DE3) pLySs cells, fusion protein was induced overnight at 15°C with isopropyld‐thio‐galactopyranoside (final concentration 0.1 mM). The cell pellets were then disrupted by means of an ultrasonic probe and centrifuged. The supernatants containing Kirola‐like protein‐6×His were purified using a His‐Accept (Nacalai Tesque) in accordance with the manufacturer's instructions, followed by further purification by ion exchange chromatography. The resulting fusion protein was verified with 96% peptide sequence coverage identified by means of an LTQ Orbitrap Discovery mass spectrometer (Thermo Fisher Scientific).

### 2.8 Protein inhibition assay

Using SDS‐PAGE, the purified kirola‐like protein was separated and transferred to polyvinylidene fluoride membranes. The SDS‐PAGE gel was then transferred to an Immobilon‐P™ polyvinylidene difluoride membrane, and immunoblotting was performed by using a semidry blotting method.^11^ Lettuce latex was preincubated for 1 h at 4°C with the sera of a negative control and patient 1 before membrane exposure. After preincubation, the sera of the negative control and patient 1 were incubated overnight with the membranes, and an enhanced chemiluminescent Western blotting substrate was then used to detect bound IgE.

## 3 RESULTS

### 3.1 Identification of antigen

**Figure 2:**
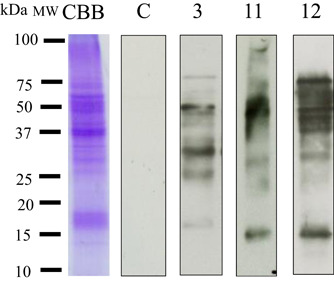
Lettuce latex IgE‐binding bands. Lane C: negative control of serum pool from a nonallergic individual; lanes 1, 2, and 3: sera of 3 of the 13 patients. MW: molecular‐weight marker (kDa); CBB: SDS‐PAGE gel of lettuce latex stained with Coomassie Brilliant Blue. IgE, immunoglobulin E; SDS‐PAGE, sodium dodecyl sulfate–polyacrylamide gel electrophoresis.

To identify the protein that causes lettuce‐associated respiratory symptoms, we previously performed immunoblotting. In brief, we combined lettuce latex juice and patient sera. As a result, we detected bands at 51 and 17 kDa (Figure 2) in three patients. In contrast, IgE‐binding bands were not observed for the sera of the controls. Therefore, we focused on the 17 kDa protein in the following experiments. Immunoblotting data from all patients with lettuce‐associated respiratory allergy are shown in Supporting Information.

### 3.2 Verification of antigen‐antibody reaction

**Figure 3:**
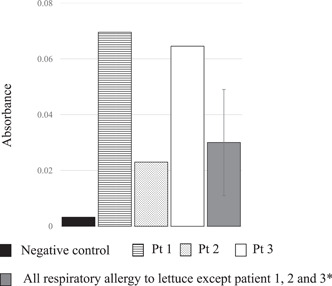
ELISA analysis of negative control, Pt 1, Pt 2, Pt 3 and entire respiratory lettuce allergies of patients. The absorbance of all respiratory lettuce allergies of patients is expressed as an average ± standard deviation. ELISA, enzyme‐linked immunosorbent assay.

To validate the antigen‐antibody reaction to lettuce latex, patient sera were treated with lettuce latex juice. As shown in Figure 3, specific IgE levels were higher for the above three patients than for the negative control. This result revealed that 3 patients were allergic to lettuce latex. Furthermore, we compared the specific IgE values of these three patients with those of the patients who did not detect the band at 17 kDa using Mann–Whitney *U* test. The specific IgE values of these thee patients tended to be higher than those of the patients who did not detect the band at 17 kDa (median: 0.065 [interquartile range {IQR}: 0.044–0.067] vs. median: 0.021 [IQR: 0.017–0.0301], *p* = .11).

### 3.3 Identification and characterization of proteins

The 17 kDa bands, which were confirmed by CBB staining, were removed from the gel and analyzed with a mass spectrometer to identify the peptide sequences of the lettuce latex allergens. NanoLC‒MS/MS was then carried out to obtain the sequences of a number of internal peptides, and proteins were identified by searching the SwissProt database with the Mascot software (http://www.matrixscience.com). Trypsin and keratin protein originating from the experimenters were eliminated, and protein identification was again carried out by searching the *L. sativa* genome database (https://www.ncbi.nlm.nih.gov/genome?term=Lactuca%20sativa) with the Mascot software. As a result of the analysis the 17 kDa protein was identified as a kirola‐like protein (Table 1).

**Table 1:** Protein identification and characterization.

Estimated molecular weight by SDS‐PAGE	17
Molecular weight of identified protein	17,329
% recognition of patient	23.1% (3/13)
Taxonomy	*Lactuca sativa*
SPROT ID	XP_023750935.1
Match score	215
Identification	kirola‐like
% of matched peptide	50%
Peptides (matched peptides)	MTLSGTLVNQ VTIKSDGDVF HEIFRQRPHH ISEMSPGCIK NVDLHEGEWG VVGSVIVWDF IHDGKAKVAK EVIEAIDEEK KSVCFKVIGG DILEAYKTFL ITVHVDTNGE ENIVTWTFHY EKVNENIDDP HTLMDFCLTV TKDIENHHLP KAN
Taxonomy: *Lactuca sativa* (45,242 sequences; 19,723,886 residues)	

Abbreviation: SDS‐PAGE, sodium dodecyl sulfate–polyacrylamide gel electrophoresis.

### 3.4 Protein purification and validation

The kirola‐like protein was purified from *Escherichia coli*. To validate the quality of this purified protein, we performed immunoblotting with this protein and sera from a negative control and patient 1. As for now, we unfortunately did not observe an inhibitory effect for this protein (data not shown).

## 4 DISCUSSION

Previously, using the sera of patients suffering from lettuce‐related respiratory allergies, we identified epidermis‐specific EP1‐like glycoprotein (51 kDa) secreted in lettuce latex to be a lettuce allergen.^1^ In the current study, we found kirola‐like protein (17 kDa) to be a novel allergen that could cause occupational respiratory allergy in farmers. The official Act d 11 allergen nomenclature indicates that kirola is a 17 kDa protein found in high quantities in ripe kiwifruit having green or yellow flesh.^13^ Ten percent of individuals are allergic to kiwifruit containing IgE that is in accordance with Act d 11.^14^ The protein is a member of the major latex protein/ripening‐related protein family (MLP/RRP) and is the first member of the family found to be an allergen.^15^

Our study is limited in certain respects. For instance, we detected a common band at 17 kDa from sera of 3 of 13 patients with occupational respiratory allergy. The 17 kDa kirola‐like protein we identified in this study is unlikely the main antigen of lettuce‐associated respiratory allergy since the percentage of patients who tested positive by immunoblotting was low. In our previous study, we demonstrated in vivo by means of skin‐prick tests and specific inhalation challenges that lettuce latex was a lettuce‐associated respiratory allergen. While 17 and 51 kDa proteins were shown to be lettuce‐associated respiratory allergy antigens in vitro, they were not observed to be so in vivo. In fact, we have not been able to prove that the 17 kDa protein purified is the antigen responsible for lettuce‐associated respiratory allergy because we have not been able to conduct challenge tests with the 17 kDa protein from an ethical standpoint.

We could not show the purified protein's inhibitory effect in the present study, partly because the amount of protein in the lettuce latex juice was originally low. Another reason may be the difference in reactivity between kirola‐like protein in lettuce latex juice and purified kirola‐like protein. We are addressing these problems, and we hope to successfully purify the novel protein that is associated with lettuce allergy in the near future.

Through identifying the 17 and 51 kDa proteins related to occupational respiratory allergies to lettuce allergens, we could contribute to the diagnosis of occupational respiratory allergies to lettuce in the future.

## 5 CONCLUSIONS

We identified a 17 kDa protein that is a potentially novel antigen of lettuce‐associated respiratory allergy.

## AUTHOR CONTRIBUTIONS

**Junya Yoshioka**: investigation; methodology; writing—original draft. **Tatsuya Nagano**: conceptualization; data curation; formal analysis; funding acquisition; investigation; methodology; project administration; writing—original draft; writing—review & editing. **Reina Sekiya**: conceptualization. **Erika Yano**: data curation. **Naoya Hatano**: methodology. **Naoko Katsurada**: data curation. **Masatsugu Yamamoto**: conceptualization. **Motoko Tachihara**: Supervision. **Yuichi Uno**: Data curation; Methodology. **Tatsuya Moriyama**: conceptualization; supervision. **Yoshihiro Nishimura**: conceptualization; project administration; supervision; validation; writing—review & editing. **Kazuyuki Kobayashi**: funding acquisition; project administration; supervision.

## CONFLICT OF INTEREST STATEMENT

The authors declare no conflicts of interest.

## Supporting information

Supporting information.

## Data Availability

Data are available from the article. If any one require, we will respond to request.

## References

[1] Sekiya R , Nagano T , Moriyama T , et al. Occupational respiratory allergy to lettuce in lettuce farmers. Clinical & Experimental Allergy. 2020;50(8):932‐941. 32542808 10.1111/cea.13682

[2] San Miguel‐Moncín M , Krail M , Scheurer S , et al. Lettuce anaphylaxis: identification of a lipid transfer protein as the major allergen. Allergy. 2003;58(6):511‐517. 12757453 10.1034/j.1398-9995.2003.00097.x

[3] Bascones O , Rodríguez‐Pérez R , Juste S , Moneo I , Caballero ML . Lettuce‐induced anaphylaxis. identification of the allergen involved. J Investig Allergol Clin Immunol. 2009;19(2):154‐157. 19476020

[4] Vila L , Sánchez G , Sanz ML , et al. Study of a case of hypersensitivity to lettuce (*Lactuca sativa*). Clinical & Experimental Allergy. 1998;28(8):1031‐1035. 9756210 10.1046/j.1365-2222.1998.00338.x

[5] Alonso MD , Martin JA , Cuevas M , et al. Occupational protein contact dermatitis from lettuce. Contact Dermatitis. 1993;29(2):109‐110. 8365175 10.1111/j.1600-0536.1993.tb03504.x

[6] Franck P , Kanny G , Dousset B , Nabet P , Moneret‐Vautrin DA . Lettuce allergy. Allergy. 2000;55(2):201‐202. 10726743 10.1034/j.1398-9995.2000.00373.x

[7] Paulsen E , Andersen KE . Lettuce contact allergy. Contact Dermatitis. 2016;74(2):67‐75. 26289653 10.1111/cod.12458

[8] Hartz C , Del mar san miguel‐Moncín M , Cisteró‐Bahíma A , et al. Molecular characterisation of lac s 1, the major allergen from lettuce (*Lactuca sativa*). Mol Immunol. 2007;44(11):2820‐2830. 17349693 10.1016/j.molimm.2007.01.030

[9] Muñoz‐García E , Luengo‐Sánchez O , Haroun‐Díaz E , et al. Identification of thaumatin‐like protein and aspartyl protease as new major allergens in lettuce (*Lactuca sativa*). Mol Nutr Food Res. 2013;57(12):2245‐2252. 23983075 10.1002/mnfr.201300139

[10] Laemmli UK . Cleavage of structural proteins during the assembly of the head of bacteriophage T4. Nature. 1970;227(5259):680‐685. 5432063 10.1038/227680a0

[11] Kyhse‐Andersen J . Electroblotting of multiple gels: a simple apparatus without buffer tank for rapid transfer of proteins from polycrylamide to nitrocellulose. J Biochem Biophys Methods. 1984;10(3‐4):203‐209. 6530509 10.1016/0165-022x(84)90040-x

[12] Moriyama T , Yano E , Kitta K , Kawamoto S , Kawamura Y , Todoriki S . Effect of gamma irradiation on soybean allergen levels. Biosci Biotechnol Biochem. 2013;77(12):2371‐2377. 24317048 10.1271/bbb.130487

[13] Ciardiello MA , Giangrieco I , Tuppo L , et al. Influence of the natural ripening stage, cold storage, and ethylene treatment on the protein and IgE‐binding profiles of green and gold kiwi fruit extracts. J Agricult Food Chem. 2009;57(4):1565‐1571. 10.1021/jf802966n 19199584

[14] Chruszcz M , Ciardiello MA , Osinski T , et al. Structural and bioinformatic analysis of the kiwifruit allergen Act d 11, a member of the family of ripening‐related proteins. Mol Immunol. 2013;56(4):794‐803. 23969108 10.1016/j.molimm.2013.07.004 PMC3783527

[15] D'Avino R , Bernardi ML , Wallner M , et al. Kiwifruit Act d 11 is the first member of the ripening‐related protein family identified as an allergen. Allergy. 2011;66(7):870‐877. 21309790 10.1111/j.1398-9995.2011.02555.x

