# Supplementary material for: Identification of a 17 kDa protein that is a potentially novel antigen of lettuce‐associated respiratory allergy in farmers
Source: Immun Inflamm Dis. 2023 Nov 20;11(11):e1093. doi: 10.1002/iid3.1093 (PMC10659754; doi:10.1002/iid3.1093)
Supplement: Supplementary file 1 — Supporting information. [file IID3-11-e1093-s001.pptx]

## Slide 1
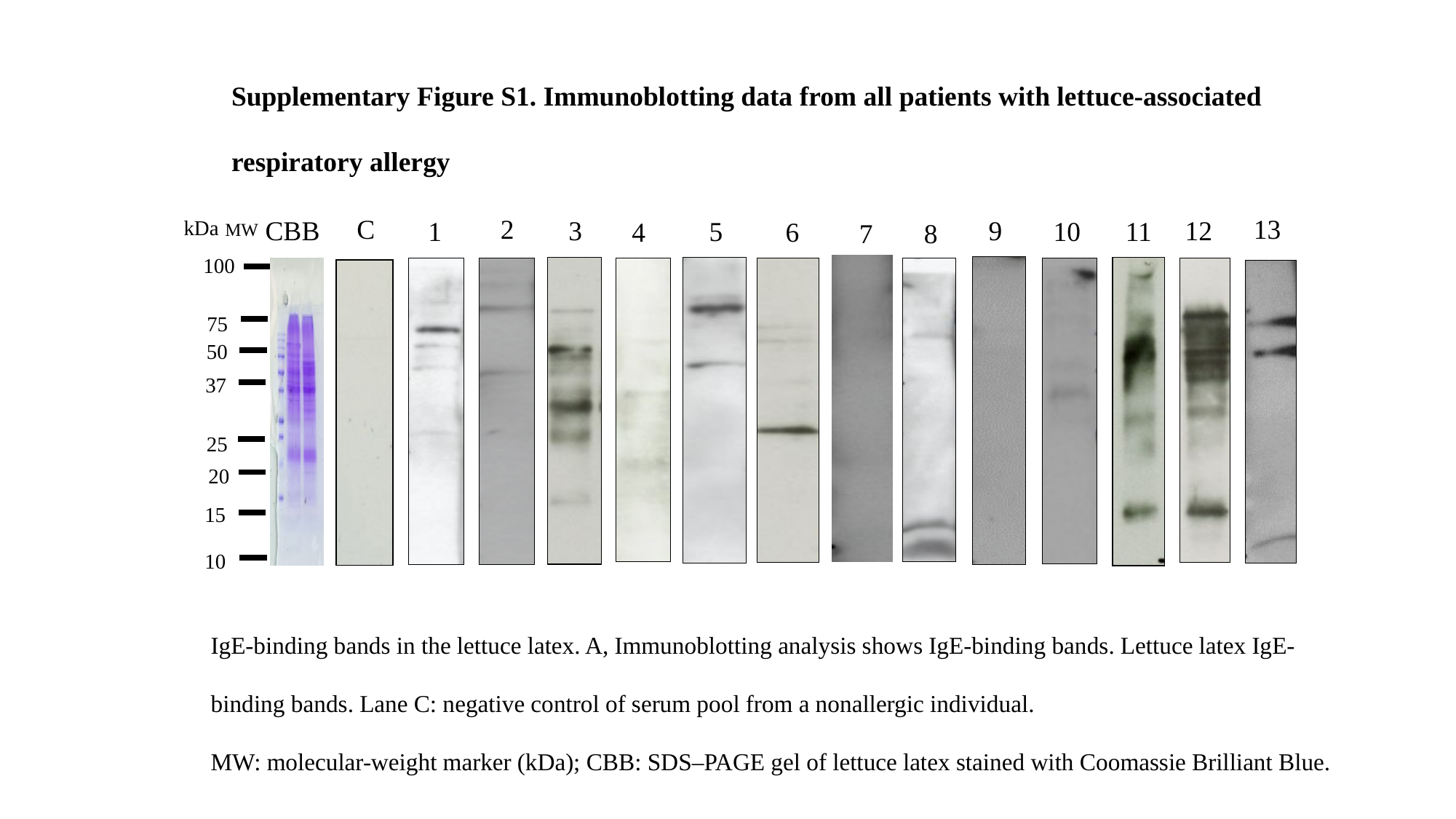

Supplementary Figure S1. Immunoblotting data from all patients with lettuce-associated respiratory allergy
C
12
CBB
3
kDa
11
MW
100
75
50
37
25
20
15
10
2
13
9
5
10
1
4
6
7
8
IgE-binding bands in the lettuce latex. A, Immunoblotting analysis shows IgE-binding bands. Lettuce latex IgE-binding bands. Lane C: negative control of serum pool from a nonallergic individual.
MW: molecular-weight marker (kDa); CBB: SDS‒PAGE gel of lettuce latex stained with Coomassie Brilliant Blue.
